# Yeast nutrient transceptors provide novel insight in the functionality of membrane transporters

**DOI:** 10.1007/s00294-013-0413-y

**Published:** 2013-10-11

**Authors:** Joep Schothorst, Harish Nag Kankipati, Michaela Conrad, Dieter R. Samyn, Griet Van Zeebroeck, Yulia Popova, Marta Rubio-Texeira, Bengt L. Persson, Johan M. Thevelein

**Affiliations:** 1 Laboratory of Molecular Cell Biology, Institute of Botany and Microbiology, KU Leuven, Leuven, Belgium; 2 Department of Molecular Microbiology, VIB, Kasteelpark Arenberg 31, 3001 Leuven-Heverlee, Flanders Belgium; 3 Department of Chemistry and Biomedical Sciences, Linnaeus University Centre for Biomaterials Chemistry, Linnaeus University, 391 82 Kalmar, Sweden

**Keywords:** Nutrient signaling, Transceptor, Co-transport, Proton binding, Endocytosis, Ubiquitination

## Abstract

In the yeast *Saccharomyces cerevisiae* several nutrient transporters have been identified that possess an additional function as nutrient receptor. These transporters are induced when yeast cells are starved for their substrate, which triggers entry into stationary phase and acquirement of a low protein kinase A (PKA) phenotype. Re-addition of the lacking nutrient triggers exit from stationary phase and sudden activation of the PKA pathway, the latter being mediated by the nutrient transceptors. At the same time, the transceptors are ubiquitinated, endocytosed and sorted to the vacuole for breakdown. Investigation of the signaling function of the transceptors has provided a new read-out and new tools for gaining insight into the functionality of transporters. Identification of amino acid residues that bind co-transported ions in symporters has been challenging because the inactivation of transport by site-directed mutagenesis is not conclusive with respect to the cause of the inactivation. The discovery of nontransported agonists of the signaling function in transceptors has shown that transport is not required for signaling. Inactivation of transport with maintenance of signaling in transceptors supports that a true proton-binding residue was mutagenised. Determining the relationship between transport and induction of endocytosis has also been challenging, since inactivation of transport by mutagenesis easily causes loss of all affinity for the substrate. The use of analogues with different combinations of transport and signaling capacities has revealed that transport, ubiquitination and endocytosis can be uncoupled in several unexpected ways. The results obtained are consistent with transporters undergoing multiple substrate-induced conformational changes, which allow interaction with different accessory proteins to trigger specific downstream events.

## Introduction

Plasma membrane transporters are the entry gate for most of the nutrients used by cells to generate energy and building blocks for cellular maintenance, growth and development. In this sense, they may exert important control over all cellular activities, either as simple providers of the nutrient molecules into the cellular interior or because they may signal the presence of the extracellular nutrients to the cellular machinery. A striking example of this sensing role of transporters has been discovered in yeast where multiple nutrient transporters appear to control activation of the protein kinase A pathway at the onset of fermentable growth induction. When yeast cells grow on a fermentable sugar, like glucose, they display high activity of the protein kinase A (PKA) pathway, causing low levels of storage carbohydrates, low stress tolerance, and high growth and fermentation rates. When such fermenting cells are starved for a single essential nutrient, like nitrogen, phosphate or sulfate, they will arrest growth, reduce their fermentation rate, accumulate high levels of storage carbohydrates and develop high stress tolerance, indicating that the activity of the PKA pathway is being downregulated (Thevelein and de Winde 1999). Re-addition of the lacking nutrient triggers rapid reversal to a high-PKA phenotype and one of the earliest read-outs is activation of the PKA phosphorylation target trehalase (Hirimburegama et al. 1992; Schepers et al. 2012). Transporters have been shown to play an essential role as receptors in this activation process: Gap1 for activation with amino acids (Donaton et al. 2003; Van Zeebroeck et al. 2009; Rubio-Texeira et al. 2012), Mep2 for ammonium (Van Nuland et al. 2006), Pho84 for phosphate (Giots et al. 2003; Popova et al. 2010) and Sul1,2 for sulfate (Kankipati et al., in preparation). Because of their double function as transporter and receptor, we have called such proteins transceptors (Holsbeeks et al. 2004).

Transporters are well known to function either as passive transport systems, carrying molecules down their concentration gradient, or as active transport systems, using energy to carry molecules uphill against their concentration gradient. Active transport is mediated by carriers which couple transport directly to the use of energy derived from hydrolysis of an ATP molecule or by carriers which make use of a pre-established electrochemical ion gradient to drive co-transport of the nutrient molecule and a co-transported ion. The latter category comprises symporters and antiporters, which carry the ion in the same or opposite direction, respectively, as the transported substrate. In recent years, much insight has been gained in the structure and functioning of transporters, due to the successes in crystallization of substrate–transporter complexes and determination of their 3D-structures, for instance LacY, GlpT, FucP and PipT. In spite of this, important questions have remained difficult to answer. One of these is the identity of the amino acid residues that are responsible for binding the co-transported ion during its passage through the transporter. Site-directed mutagenesis of candidate residues, that can bind a co-transported ion and are located in or close to a transmembrane domain, would normally be the method of choice. However, replacement of such residues can abolish ion coupling in transport but can also affect the whole functionality of the transporter, because the residue is in some way important for maintenance of the proper structure, for insertion in the membrane or for another feature of the transporter that is essential for transport. This makes it difficult to draw a definite conclusion about the precise role of the residue. The discovery of the yeast transceptors, in which the signaling function was found to be independent from the transport function, provides a novel approach to assess the general functionality of the transporter and may therefore help in providing evidence that a certain amino acid residue is truly involved in binding the co-transported ion.

Substrate-induced endocytic internalization and sorting to the vacuole is a well-known mechanism by which cells regulate the level of transporter in the plasma membrane as a function of external substrate availability. The prevailing idea in the literature is that transport of the substrate through the transporter generates in some way a signal and/or makes the transporter susceptible for ubiquitination (Cain and Kaiser 2011; Seron et al. 1999; Gournas et al. 2010; Liu and Culotta 1999; Jensen et al. 2009; Felice et al. 2005). The latter is then thought to serve as the signal for endocytosis, which is followed by sorting and breakdown of the transporter in the vacuole (Dupre et al. 2004; Gitan and Eide 2000; Liu et al. 2007; Hicke and Dunn 2003; Nikko et al. 2008; Eguez et al. 2004; Lauwers et al. 2010; Horak 2003; Shih et al. 2000; Barberon et al. 2011).

The compounds that we have developed to study the relationship between transport and signaling in the Gap1 transceptor have now been used as novel tools to study the connection between the different events happening in substrate-induced internalization of the transceptors. This has led to the unexpected finding that these events can be uncoupled in several previously unanticipated ways.

## Identification of amino acid residues involved in binding co-transported ions

### Proton-coupled transporters

Identification of proton-binding residues in a few model transporters has been inferred mainly from site-directed mutagenesis, crystal structure determinations and the suggested modus operandi of the transporter. The modus operandi of individual protons in a proton-driven uptake system can be dissected in three related aspects: (1) coupling between substrate binding/dissociation and conformational changes in the transporter; (2) coupling between the substrate binding/dissociation and protonation/deprotonation of residues; and (3) coupling between protonation/deprotonation and conformational changes in the transporter.

Translocation of protons involves protonation/deprotonation of certain amino acid residues. Most frequently these are Glu/Asp/His residues, and to a lesser extent Lys/Arg/Tyr. The lactose permease of *Escherichia* *coli*, LacY, serves as a paradigm when assessing proton-driven transport (Abramson et al. 2004). A tightly interconnected hydrogen bond and salt bridge cluster composed of Glu325, Lys319 and His322 (TM10), Arg302 (TM9), Glu269 (TM8), and Tyr236 and Asp240 (TM7), can be found in the crystal structure. Glu325, His322 and Arg302 are thought to be directly involved in proton translocation (Kaback et al. 2001). Due to the lack of a potential hydrogen bond donor in the immediate vicinity, Glu325 is believed to be protonated, and thus prevents H^+^ escape from the cluster, maintaining coupling with the sugar-binding site (Smirnova et al. 2009). The involvement and importance of protons can be seen in a brief description of what is believed to be the main transport mechanics: (1) the Co-apo conformation is immediately protonated. The H^+^ is shared by Glu269 and His322; (2) the substrate is initially recognized by Trp151, Arg144 and Glu126. This will lead to a disruption of the salt bridge between Arg144 and Glu126, bringing His322 in contact with Glu325. This may induce proton transfer from His322 to Glu325, leading to a rapid conformational change and to the cytoplasm facing conformation, Ci; (3) the substrate is released into the cytoplasm; (4) the salt bridge between Arg144 and Glu126 is re-established. H^+^ is released from Glu325. It has been suggested that Arg302 could interact with Glu325 to drive proton release from Glu325 because mutants in either residue exhibit the same specific defect in proton-coupled lactose translocation reactions, with no effect on sugar binding, exchange or counterflow (Sahin-Toth and Kaback 2001).

A similar protonation/deprotonation mechanism has been proposed to play an important role in proton-dependent oligopeptide transporter (POT) family members. The crystal structures of oligopeptide transporters from the bacteria *Shewanella oneidensis* (PepT_So_) (Newstead et al. 2011), *Streptococcus thermophilus* (PepT_St_) (Solcan et al. 2012) and *Geobacillus kaustophilus* (GkPOT) (Doki et al. 2013) have been reported. They have suggested mechanisms involved in proton-coupled peptide transport. In case of the GkPOT, the positive charge of Arg43 may facilitate the deprotonation of Glu310 during the transition to the occluded, apo state, which allows the formation of a salt bridge between Arg43 and Glu310. Following the protonation of Glu310, the substrate will bind to Arg43 and Glu310. Mutagenesis of Glu310 to Gln resulted in an inactive transporter, suggesting that Glu310 is involved in H^+^ binding/translocation, by blocking the transition of the transporter between the inward- and outward-open states (Doki et al. 2013). Furthermore, Glu32 has been proposed to be of importance (based on molecular dynamics simulations) in the transition mechanism between outward-facing and occluded states. It has been proposed that Glu32 is another protonation site, and the H^+^ translocation occurs between Glu32 and Glu310 (similar to the H^+^ translocation from His322-Glu269 to Glu325 in LacY). For the human POT members PepT1 and PepT2, His57 and His87, respectively, have been suggested as primary protonation sites based on results of site-directed mutagenesis (Fei et al. 1997; Uchiyama et al. 2003), whereas in case of the PepT_So_ His61 was suggested to be the primary protonation site based on the crystal structure determination (Newstead et al. 2011).

The proposed mechanism for fucose:H^+^ transport is also believed to involve residues that undergo a protonation/deprotonation cycle. Along the transport pathway residues Asp46 and Glu135 (3rd helical turn of TM1 and TM4, respectively) are thought to be involved in this protonation/deprotonation cycle. Transport studies, involving site-directed mutagenesis, have confirmed that these residues play an important role. Asp46 is believed to be essential for proton-dependent active transport, whereas Glu135 might be involved in substrate recognition (Dang et al. 2010). Moreover, the proposed mechanism and involvement of these two residues is as follows: (1) in the Co-apo conformation, l-fucose can only bind following protonation of Asp46. This protonation step will neutralize the negative charge and thus lower the energy barrier for the l-fucose entry/transport; (2) proton translocation from Asp46 to Glu135 will result in abolishment of the hydrogen bond between Glu135 and Tyr365 enabling binding of the substrate with the protonated Glu135; (3) the protonation and binding of the substrate to Glu135 will trigger rigid body rotation of the N- and C-domains, resulting in the Ci conformation of the transporter. The transport cycle is completed with the deprotonation of Glu135 and the release of the substrate into the cytoplasm.

**Fig. 1:**
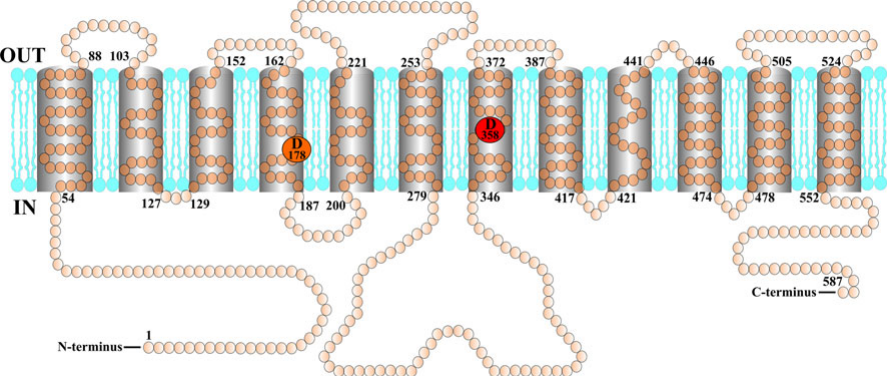
Model of the Pho84 phosphate transceptor with two putative proton-binding residues allowing uncoupling of transport and signaling. The protein has 12 predicted transmembrane domains and functions as a phosphate/proton symporter. D178 and D358 are two conserved putative proton-binding residues, predicted to be located adjacent to the phosphate translocation pathway. Mutagenesis of D178 to E or N reduces *V*_max_ with about 50 %, but does not lower signaling as measured by phosphate-induced activation of the PKA target trehalase. Mutagenesis of D358 to N strongly reduces transport to ≤20 % but does not affect signaling. Mutagenesis of D358 to E completely abolishes transport but only causes a 50 % drop in signaling

In the case of the *P. indica* phosphate transporter, (PiPT), the crystal structure was resolved with inorganic phosphate bound (Pedersen et al. 2013). Here, Asp324 is proposed to be protonated, which gives preference to phosphate binding. In case of the *Saccharomyces cerevisiae* high-affinity inorganic phosphate transporter, Pho84, site-directed mutagenesis studies have shown that Asp358 may play a role in proton-coupled phosphate transport activity (Samyn et al. 2012) (Fig. 1). Pho84 has been shown to be a transceptor for activation of the PKA pathway and previous work has shown that transport of substrate is not required for the induction of signaling by the receptor function of Pho84 (Popova et al. 2010). Interestingly, mutagenesis of Asp358 in Pho84 abolished transport but left signaling largely unaffected (Samyn et al. 2012). This shows that mutagenesis of Asp358 does not significantly compromise proper membrane insertion and general functionality of Pho84, reinforcing the suggestion from the site-directed mutagenesis that Asp358 is specifically required for proton binding. In addition, it confirmed that substrate transport is not required for signaling by the Pho84 transceptor. A similar result has been obtained, recently, for the Sul1 and Sul2 sulfate transporters. Site-directed mutagenesis of a putative proton-binding residue abolished transport without affecting signaling. This supports a role for this residue in proton binding, shows that also in this case substrate transport is not required for signaling and provides a strong argument that Sul1,2 function as sulfate transceptors (Kankipati et al., in preparation). Mutagenesis of Asp178 in Pho84 also allowed partial uncoupling of transport and signaling (Samyn et al. 2012) (Fig. 1). This has also been achieved for the Gap1 transceptor by deletion of Nhx1 or Pmp3, two Gap1-interacting proteins. Deletion of Nhx1 or Pmp3 strongly reduced amino acid uptake by Gap1, but did not affect signaling at all (Van Zeebroeck et al. 2011). All these data support that the transport and signaling functions of the transceptors are not dependent on each other. Hence, site-directed mutagenesis of putative residues involved in binding the co-transported ion can be a very efficient approach to separate the signaling from the transport function and in this way establish the capacity of a transporter to function as a transceptor.

Amino acid residues with a negatively charged side chain also play a role in the transport mechanism of yeast mitochondrial carriers (Kunji and Robinson 2010). Again, the most common residues found in the substrate-binding site are E, and also D. These residues are D130 in the yeast phosphate carrier (Phelps and Wohlrab 1991), E600 in the yeast aspartate/glutamate carrier (Cavero et al. 2003), E91 in the yeast oxodicarboxylate carrier (Palmieri et al. 2000) and E24 in the yeast GTP/GDP carrier (Vozza et al. 2004). Despite belonging to the class of sodium-dependent secondary transport proteins, the plasma membrane glutamate transporters are somewhat odd class members. These transporters utilize the downhill movement of Na^+^ and K^+^ ions to transport glutamate (Kanner and Bendahan 1982), but in addition, it has been suggested that protons are co-transported together with glutamate (Zerangue and Kavanaugh 1996). In case of the excitatory amino acid carrier (EAAC1), glutamate can only bind to the protonated form of the transporter, and upon translocation the released glutamate and H^+^ will cause relocation of its binding sites to the basic, unprotonated state (Watzke et al. 2000). Site-directed mutagenesis and kinetic measurements support the conclusion that E373 serves as a proton acceptor (Grewer et al. 2003). Furthermore, initially H295 was thought to be involved also in proton co-transport. This notion has been refuted since the replacement of H295 with glutamine, an amino acid residue that cannot be protonated, generates a fully functional transporter with transport kinetics that are close to those of the wild-type EAAC1. H295 is now deemed to play a rather secondary role as protonation-dependent modulator, where the protonated H295 dramatically decreases the affinity of the transporter for glutamate (Tao and Grewer 2005).

### Na-coupled transport

The melibiose permease of *E. coli* accomplishes uphill transport by utilizing Na^+^, Li^+^ or H^+^ as cosolute (Tsuchiya et al. 1985; Pourcher et al. 1995). Several mutagenesis studies have shown that residues D19, D55, D59 and D124 are important for Na^+^-dependent affinity and transport of melibiose (Pourcher et al. 1991, 1993; Zani et al. 1993; Wilson and Wilson 1992). More recently, substrate-induced IR_diff_ spectroscopy was applied to reevaluate the importance of these four residues (Granell et al. 2010) and their involvement in Na^+^-binding/interaction. The results suggested that only D55 and D59 are essential ligands for Na^+^, since only the D55C and D59C mutants do not exhibit any structural variation upon incubation with Na^+^.

Several transporters, which were originally not assigned to sequence-related families, have in common the LeuT-like fold. To date, from the eight transporters having the LeuT-like fold (Perez and Ziegler 2013), a detailed description of the alternating-access mechanism and the Na^+^-binding sites for the LeuT from the neurotransmitter:sodium symporter (NSS) family (Weyand et al. 2008), Mhp1 from the nucleobase:cation symporter-1 (NCS1) family (Krishnamurthy and Gouaux 2012; Krishnamurthy et al. 2009) and BetP from the betaine/carnitine/choline transporter (BCCT) family (Perez et al. 2012) has been made possible by combining structural, computational and biophysical approaches. Based on the crystal structure and molecular dynamics analysis, the LeuT from *Aquifex aeolicus* reveals two sodium-binding sites, namely Na1 and Na2. The Na^+^ in Na1 is coordinated by five residues, namely A22 (TM1), T254 (TM6), N27 (TM1), N286 (TM7) and T254 (TM6). The coordination of the Na^+^ in Na2 is executed by G20 (TM1), V23 (TM1), A351 (TM8), T354 (TM8) and S355 (TM8).

In conclusion, putative amino acid residues responsible for the binding of co-transported ions have generally been suggested on the basis of site-directed mutagenesis studies of candidate residues located in or close to transmembrane domains. However, this is not sufficient to make a definite conclusion since replacement of an amino acid residue can affect transport activity also because it disturbs the general structure or functioning of the transporter. Advanced biophysical techniques are generally required to gain additional evidence for a role of a specific residue in binding the co-transported ion.

## Relationship between signaling, transport, ubiquitination and endocytosis

A major breakthrough in transceptor analysis was the identification of nontransported substrate analogues that were able to trigger the signaling function of the transceptor: l-Leu-Gly for Gap1 (Van Zeebroeck et al. 2009), glycerol-3-phosphate and other organic phosphate esters for Pho84 (Popova et al. 2010) and d-glucosamine 2-sulfate for Sul1,2 (Kankipati et al., in preparation). These molecules provided first of all a major new argument for the receptor function of the transporters. With these nontransported signaling agonists, the transceptors function as pure receptors. In addition, the compounds turned out to be interesting new tools for studying the signaling function of the transceptors independently of their transport function, but have also turned out to be powerful tools to investigate other outstanding questions with respect to transporter functionality and regulation.

**Fig. 2:**
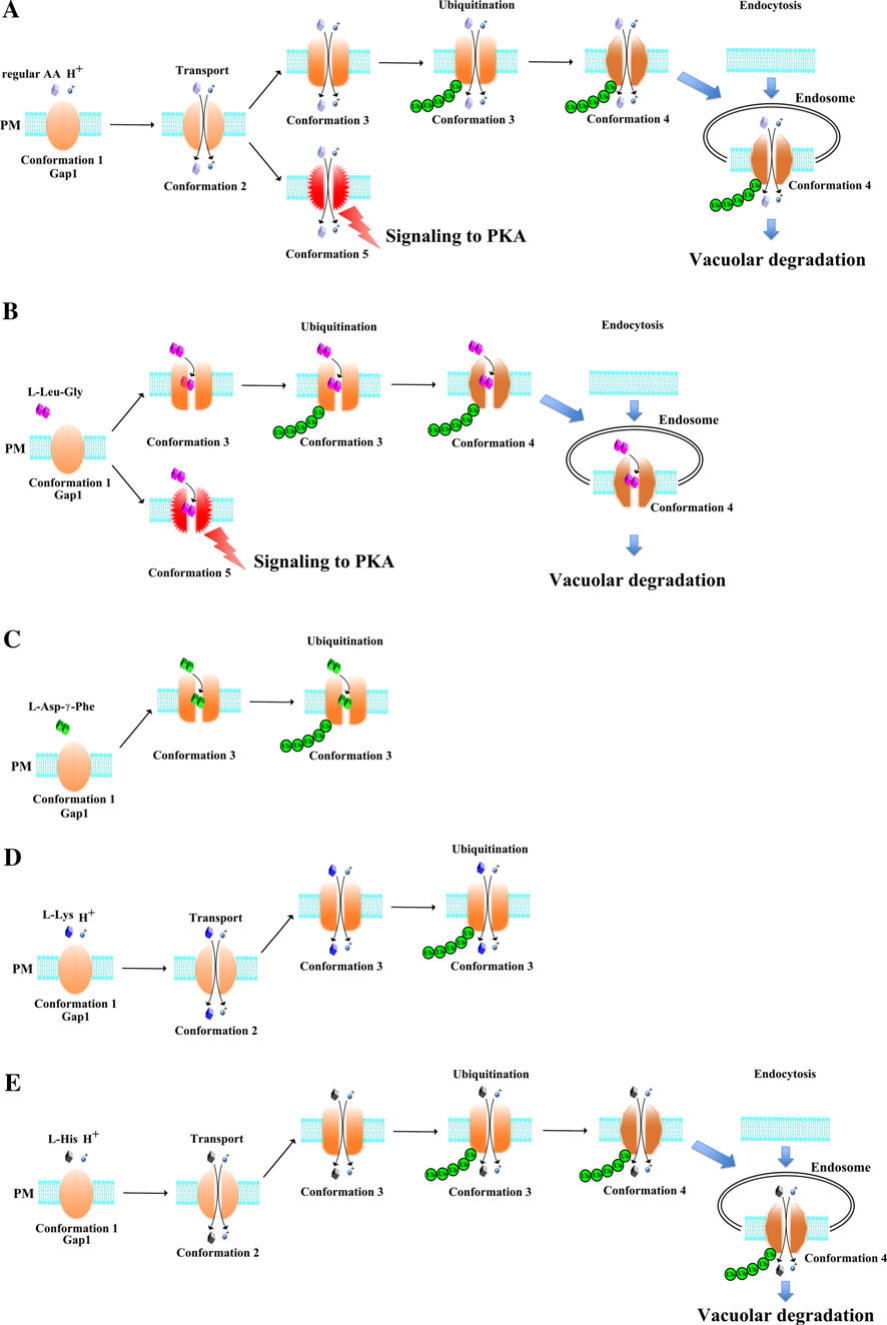
Specific compounds allow uncoupling of transport, ubiquitination, endocytosis and signaling in the Gap1 transceptor. **a** The uptake of an amino acid triggers a (series of) conformational changes, of which one allows the ubiquitination of Gap1. Ubiquitination and an additional conformational change are required to trigger endocytosis of Gap1. Signaling to PKA is triggered by an independent conformational event. **b** The dipeptide l-Leu-Gly is not transported but is able to trigger all subsequent events like a regular amino acid. **c** The dipeptide l-Asp-γ-Phe is also not transported but is only able to trigger the conformation that allows ubiquitination. **d** l-Lysine is transported but is not able to trigger signaling nor endocytosis. However, it can also induce the conformation that elicits ubiquitination. **e** l-histidine cannot trigger signaling but can induce the whole endocytic internalization process just like a regular amino acid

The molecular mechanisms underlying substrate-induced endocytosis of nutrient transporters have been studied in great detail in yeast, with Gap1 serving as the main model system (Jauniaux and Grenson 1990; Lauwers et al. 2010; Magasanik and Kaiser 2002; Chen and Kaiser 2002) (Fig. 2a). The first well-established change in the Gap1 permease following addition of amino acid is its ubiquitination, which is followed by sorting to the multivesicular body (MVB) and degradation in the vacuole/lysosome. Ubiquitination is accomplished by the E3 ubiquitin ligase Rsp5 (Soetens et al. 2001). Prevention of ubiquitination by mutagenesis of the N-terminal Lys 9 and Lys 16 residues, abolishes endocytosis, which has been taken as evidence that ubiquitination serves as a signal for endocytosis. This reasoning has been extended to many other transporters and ubiquitination is generally conceived as the main signal triggering endocytosis (Barberon et al. 2011; Shih et al. 2000; Horak 2003; Lauwers et al. 2010; Eguez et al. 2004; Nikko et al. 2008; Hicke and Dunn 2003; Liu et al. 2007; Gitan and Eide 2000; Dupre et al. 2004).

The initial trigger for recruitment of the Rsp5 ubiquitin ligase is not well understood, but is generally thought to be caused in some way by the transport of the substrate through the carrier. This has been concluded from the behavior of mutant forms of the transporters that displayed strongly reduced uptake capacity and were no longer endocytosed. Such results have been obtained for the Smf1 metal transporter (Liu and Culotta 1999; Jensen et al. 2009), the Fur4 uracil permease (Seron et al. 1999), the Pho84 phosphate transporter (Petersson et al. 1999; Lundh et al. 2009), the Ftr1 iron transporter (Felice et al. 2005), Gap1 (Cain and Kaiser 2011) and the sulfate transporter Sul2 (Jennings and Cui 2012) in *S. cerevisiae* and for the uric acid/xanthine transporter, *An*UapA, in *Aspergillus nidulans* (Gournas et al. 2010). For the latter transporter, 3-methylxanthine, was identified as a competitive inhibitor of transport, unable to induce endocytosis. This was taken as evidence that interaction of the substrate with the *An*UapA transporter was not enough to trigger endocytosis and that transport was required. The possibility that a substrate could be transported through a nutrient permease without triggering endocytosis has apparently never been considered.

The discovery of the nontransported signaling agonist l-Leu-Gly indicated that this compound physically interacted with Gap1, which raised the question whether it would also be able to trigger endocytosis. Interestingly, l-Leu-Gly triggered ubiquitination and endocytosis in a similar way as regular amino acids (Van Zeebroeck et al., in preparation) (Fig. 2b). This indicates for the first time that a full transport cycle is not required to trigger endocytosis. Classical receptors are well known to undergo ligand-induced endocytosis (Sorkin and Von Zastrow 2002) and transceptors thus behave for substrate-induced endocytosis in a similar way as classical receptors, as was also observed for signaling. This emphasizes again the apparent similarity between the behavior of transceptors and classical receptors (Kriel et al. 2011). Also in mammalian cells, the surprising similarity between the mechanisms involved in substrate-induced transporter endocytosis and ligand-induced receptor endocytosis has been pointed out (Miranda and Sorkin 2007).

The dipeptide l-Leu-Gly is a competitive inhibitor of Gap1 transport. The same is true for l-Asp-γ-Phe, but this dipeptide is unable to trigger signaling. It was also unable to trigger endocytosis, but unexpectedly induced oligo-ubiquitination (Van Zeebroeck et al., in preparation) (Fig. 2c). This provides the first indication that oligo-ubiquitination of a transporter may not be enough to trigger its endocytosis and that therefore an additional event is required.

An unexpected discovery was also that some amino acids, l-lysine, l-histidine and l-tryptophan, are very well transported by Gap1 but they are not able to trigger signaling (Van Zeebroeck et al. 2009). Examination of ubiquitination and endocytosis with these amino acids unexpectedly revealed that l-lysine transport leads to ubiquitination but not endocytosis (Fig. 2d). l-lysine even counteracts endocytosis triggered by a regular amino acid, like l-citrulline. l-histidine, on the other hand, triggered efficient ubiquitination and endocytosis (Van Zeebroeck et al., in preparation) (Fig. 2e). These results demonstrated that signaling, ubiquitination and endocytosis can be uncoupled in different ways. There is no evidence that endocytosis can happen without ubiquitination, since Gap1 mutated in the N-terminal lysines 9 and 16, that function as ubiquitin attachment sites, is completely deficient in endocytosis (Soetens et al. 2001). Hence, ubiquitination is essential but apparently not sufficient for endocytosis.

These new results on substrate-induced signaling, ubiquitination and endocytosis of transceptors may also indicate that different substrates follow a somewhat different passageway when carried through the transporter, and/or that they trigger different conformational changes during their passage. Alternatively, the different substrates may trigger the same series of conformational changes but with different kinetics. To trigger a downstream event, a specific conformation would have to persist long enough to allow proper interaction with a signal transmission protein or a protein that in some way supports initiation of ubiquitination or endocytosis.

## Transceptors and substrate-induced internalization of transporters in other organisms

Several examples have been reported where transporters appeared to carry out an additional regulatory function triggered by sensing of the nutrient (Gojon et al. 2011; Hundal and Taylor 2009; Rogato et al. 2010; Stolarczyk et al. 2010; Hyde et al. 2007; Goberdhan et al. 2005). However, as opposed to the situation with the yeast transceptors controlling activation of the PKA pathway, no common principles, either for mechanisms involved or targets affected, have been identified. In spite of this, the use of substrate analogues may turn out to be a very useful approach to gain first of all strong evidence for the presence of an additional receptor function in the transporter and second to learn about the mechanisms involved in the signaling function.

Substrate-induced internalization of nutrient transporters has also been documented in other organisms. As mentioned previously, detailed studies have been made on the uric acid/xanthine transporter, *An*UapA, in *A. nidulans* (Gournas et al. 2010; Diallinas 2013). Also in this case, substrate analogues have been used to gain insight in the underlying mechanisms. In mammalian cells, evidence has also been obtained for ubiquitination as a signal for the initiation of substrate-induced endocytosis in several types of nutrient transporters (Melikian 2004; Zahniser and Sorkin 2009; Vina-Vilaseca et al. 2011; Miranda et al. 2007).

## Conclusions

The discovery of a nutrient receptor function in a set of yeast plasma membrane transporters that are induced by starvation for their substrate is providing unexpected insight and new approaches and tools to study the mechanisms involved in transport and regulation of the transporter protein level in the membrane. It can be expected that further elucidation of the mechanisms involved in signaling by these transceptors and their connection with transceptor downregulation will provide further unanticipated findings that would have been overlooked if the proteins would just have been studied as transporters.
